# Outcomes of combined endoscopic vitrectomy and posteriorly placed glaucoma drainage devices in pediatric patients

**DOI:** 10.1186/s12886-022-02373-3

**Published:** 2022-04-01

**Authors:** A Jacobson, CG Besirli, BL Bohnsack

**Affiliations:** 1 Kellogg Eye Center, Department of Ophthalmology and Visual Sciences, University of Michigan, 1000 Wall Street, Ann Arbor, Michigan 48105 USA; 2 Department of Ophthalmology, Northwestern University Feinberg School of Medicine, 645 N. Michigan Ave, #440, Chicago, Illinois 60611 USA; 3 Division of Ophthalmology, Ann & Robert H. Lurie Children’s Hospital of Chicago, 225 E. Chicago Ave, Box 70, Chicago, Illinois 60611 USA

**Keywords:** Pediatric glaucoma, Peters Anomaly, Sclerocornea, Microphthalmia, Glaucoma drainage device, Endoscopic vitrectomy

## Abstract

**Background:**

This study aims to describe outcomes of posteriorly-placed glaucoma drainage devices (GDD) with concurrent endoscopic vitrectomy in pediatric patients with glaucoma and corneal opacification.

**Methods:**

This retrospective case series identified patients under 18 years of age who underwent posteriorly-placed GDD implantation with concurrent endoscopic vitrectomy between 2012 and 2021. Data collected included ocular diagnoses, prior intraocular surgeries, type and position of GDD, surgical complications, and additional surgeries. Preoperative and final visual acuity, intraocular pressure (IOP), number of glaucoma medications, and exam findings were also recorded. Surgical data included type and position of GDD, Success was defined as IOP between 5-21 mmHg without visually devastating complication or need for additional glaucoma surgery.

**Results:**

Ten patients (14 eyes) with sclerocornea (6), Peters Anomaly (4), corneal decompensation from increased IOP (3), and corneal scar (1) underwent combined endoscopic vitrectomy with posteriorly-placed GDD (Baerveldt (10 eyes), Ahmed (4 eyes)) at 4.6 ± 5.8 years of age. Four eyes of 3 patients remained successful at final follow-up, while 10 eyes of 7 patients required 2.4 ± 1.3 additional surgeries for glaucoma (7) or hypotony (3). Kaplan Meier analysis demonstrated 1- and 2-year survival rates of 36% and 18%, respectively. At final follow-up (3.7 ± 2.4 years), after an average of 4.4 ± 2.4 glaucoma surgeries, 13 of 14 eyes had obtained IOP control on significantly fewer (*p*<0.0001) IOP-lowering medications. Additional complications included retinal detachment (2), chronic corneal graft failure (2), phthisis (1) and band keratopathy (1).

**Conclusions:**

Management of glaucoma in pediatric eyes with corneal opacification is challenging and often requires multiple surgeries. A combined endoscopic vitrectomy and posteriorly placed GDD is a viable technique to establish aqueous humor outflow. Although the success rate is low, this surgical approach may be useful in ultimately obtaining IOP control and preserving vision in these complex eyes.

**Supplementary Information:**

The online version contains supplementary material available at 10.1186/s12886-022-02373-3.

## Background

Glaucoma drainage devices (GDD) are a well-established treatment for medication-refractory glaucoma in children [1–4]. Tubes are typically placed in the anterior chamber due to ease of placement and good visualization at the slit lamp. However, in diseases with shallow or disorganized anterior segments, such as microphthalmia, persistent fetal vasculature, Peters Anomaly, and Axenfeld-Rieger syndrome, tube placement in the anterior chamber may not be advisable [5, 6]. Furthermore, in children and young adults, anterior rotation of the tube together with prolonged exposure and irritation of the corneal endothelium, makes posterior placement in the sulcus or pars plana a favorable alternative in pseudophakic and aphakic eyes [7, 8]. This approach may require prior or concurrent pars plana vitrectomy in order to reduce the risk of tube blockage by vitreous [5, 6]. In addition, there is a subgroup of these complex eyes that have corneal opacification that limits the feasibility of standard wide-angle pars plana vitrectomy. Endoscopy has previously been demonstrated as a viable alternative, allowing for visualization of the posterior segment despite anterior opacities in both children and adults [9–11]. While endoscopic vitrectomy combined with GDD placement has been described in adults, there is a paucity of data regarding this technique in children [12, 13]. Thus, we report our experience with this surgical technique, which to the best of our knowledge, is the largest cohort of pediatric patients to date.

## Methods

A retrospective case series identified patients 18 years of age or younger who underwent endoscopic pars plana vitrectomy combined with posteriorly-placed GDD at the University of Michigan between January 2012 and January 2021. This study was approved by the Institutional Review Board at the University of Michigan and adhered to the tenets of the Declaration of Helsinki. As a retrospective study, obtaining informed consent from the patients was waived by the Institutional Review Board at the University of Michigan. Data collection was de-identified and HIPAA compliant.

Surgical data collected included age at the time of surgery, ocular diagnoses, previous intraocular surgeries, surgical procedure details, and complications. Preoperative (last recorded before surgery) and final exam details included best-corrected visual acuity (BCVA), intraocular pressure (IOP), slit lamp findings, and number of glaucoma medications (oral and topical). IOP was measured by Icare (Revenio, Vantaa, Finland), Tono-pen (Reichert, Depew, NY) or Goldmann applanation. Change in BCVA was defined as 2 or more line difference in optotype testing or a change between the ability to count fingers, detect hand motion, and distinguish light.

The primary outcome measure was GDD success. Success was defined as IOP between 5 and 21 mmHg, no visually devastating complication, and no additional IOP-related surgery. The secondary outcomes were IOP at final follow-up and change in BCVA between final follow-up and initial presentation.

All GDD implantations were performed under general anesthesia by the same surgeon (BLB) as previously described [3, 14]. GDDs included Baerveldt 101-350 (BV350, Abbott Medical Optics, Santa Ana, CA), Baerveldt 101-250 (BV250, Abbott Medical Optics), Ahmed FP7 (New World Medical, Rancho Cucamonga, CA), and Ahmed FP8 (New World Medical). Selection of the GDD depended on the preoperative IOP, eye size, age, and previous ocular history (Supplemental Table 1). The BV350 was the preferred GDD with its large plate size and lower risk of hypertensive phase and encapsulation compared to Ahmed GDDs [15–17]. For smaller eyes, the plate of the GDD was trimmed along the posterior edge [18]. A BV250 was used in eyes too small for a trimmed BV350. Since BV350 and BV250 GDDs are valveless and require 3-6 weeks before achieving pressure-lowering effect, Ahmed FP7 and FP8 devices were used in eyes in which immediate IOP control was needed due to advanced optic nerve cupping, rapid axial lengthening, and concern for further corneal damage, despite the increased risk of the hypertensive phase and plate encapsulation. All vitrectomies were performed under general anesthesia by a single surgeon (CGB) concurrent with GDD implantation.

The surgical technique employed was previously described in Ozgonul et al. [6] Briefly, for BV350 and BV250 devices, a 5-0 polypropylene suture was placed as a ripcord within the plate and tube and then a 6-0 polyglactin suture was used to ligate the tube. In some cases, placement of a BV250 or BV350 device was performed in a staged procedure such that the plate was placed in one surgery and the tube was implanted within the eye in a second surgery at least 1 month later. For Ahmed FP7 and FP8 devices, the valve was primed with balanced salt solution. In the majority of cases, a limbal based approach was used. Following conjunctival and Tenon’s incisions, the plate was secured to the sclera with 8-0 nylon sutures. The tube was then tucked under the eyelid speculum for the vitrectomy. Twenty-three gauge trocars were placed inferotemporally, superotemporally and superonasally 0.5 to 2.0 mm posterior to the limbus. Trocar placement was adjusted based on age, eye size, and underlying eye disease. The Constellation Vitreoretinal Surgical System (Alcon Laboratories Inc, Fort Worth, TX) and the Endo Optiks (BVI Medical, Little Silver, NJ) was used to perform a core vitrectomy. The 23-gauge endoscope was standardly used due to ease of placement through the trochars. In select cases, a 23-gauge trocar was removed and the sclerostomy was enlarged to employ the 20-gauge endoscope for better resolution and visualization. A lensectomy was performed in phakic eyes. The posterior hyaloid membrane and cortical vitreous were dissected from the macula using aspiration. Peripheral vitreous was shaved with close attention to the quadrant of tube placement. At the conclusion of the vitrectomy, the GDD tube was trimmed, inserted into the appropriate sclerotomy, and secured to sclera using 9-0 nylon suture. The endoscope was then re-inserted to confirm proper positioning within the pars plana or pars plicata and to insure no remnant vitreous near the tube. A scleral patch graft was then placed over the tube and secured using 8-0 polyglactin sutures. Tenon’s capsule and conjunctiva were closed in a bilayer fashion with running 8-0 polyglactin sutures.

For statistical analyses all tests including Wilcoxon Rank Sum test (comparison between the two groups and pre- and post-operative values) and Kaplan-Meier survival curves with Log-rank (Mantel-Cox) test and accompanying 95% confidence intervals (CI) were performed with SPSS (SPSS Inc, Chicago, IL). All tests were 2-sided, and p-values less than 0.05 were considered statistically significant.

## Results

Fourteen eyes of 10 pediatric patients underwent an endoscopic vitrectomy with concurrent posterior placement of a GDD. All eyes had corneal opacification and glaucoma due to (Table 1) microphthalmia with sclerocornea (5 eyes of 3 patients), Peters Anomaly (4 eyes of 3 patients), primary congenital glaucoma with corneal decompensation (2 eyes of 1 patient), glaucoma following cataract surgery (persistent fetal vasculature) with corneal decompensation (1 eye of 1 patient), anterior segment dysgenesis with sclerocornea (1 eye of 1 patient), and corneal scar from trauma (1 eye of 1 patient).

**Table 1:** Patient Surgical Information. Demographic and surgical information for the 14 eyes of 10 patients which underwent combined endoscopic vitrectomy with posteriorly placed glaucoma drainage device

**Pt**	**Eye**	**Diagnosis**	**Prior Surgeries**	**Age (Yrs)**	**Preop Lens**	**GDD**	**Plate**	**Concurrent Surgeries**	**Follow-Up (Yrs)**
**1**	Right	Microphthalmia Sclerocornea	360º CTLC (4 treatments)	13.3	Congenital Aphakia	BV350	ST		3.7
	Left	Microphthalmia Sclerocornea	360º CTLC (4 treatments)	13.5	Congenital Aphakia	BV250	ST		3.6
**2**	Right	PCG Corneal Decompensation	180º Trabeculotomy x 2	0.1	Phakic	BV250	ST	ECCE	6.3
	Left	PCG Corneal Decompensation	180º Trabeculotomy x 2	0.1	Phakic	BV250	ST	ECCE	6.4
**3**	Left	Microphthalmia Sclerocornea	FP7 Placement PKP ECCE	0.7	Aphakic	BV350	ST	FP7 Explantation	6.6
**4**	Right	Peters Anomaly	None	2.8	Phakic	BV350	ST	ECCE	4.2
	Left	Peters Anomaly	270º CTLC (4 treatments)	2.5	Phakic	BV350	ST	ECCE	4.6
**5**	Right	Peters Anomaly	180º Trabeculotomy	0.1	Phakic	FP7	SN	ECCE	4.3
**6**	Right	Peters Anomaly	PKP	0.5	Phakic	BV250	ST	ECCE	2.2
**7**	Right	Microphthalmia Sclerocornea	270º CTLC (2 treatments)	2.4	Congenital Aphakia	FP8	ST		0.79
	Left	Microphthalmia Sclerocornea	270º CTLC (2 treatments)	2.4	Congenital Aphakia	FP8	ST	Cryotherapy for vitreous tag	0.75
**8**	Right	PFV Microphthalmia Glaucoma Following Cataract Surgery Corneal Decompensation	180º CTLC (1 treatment) ECCE	1.4	Aphakic	FP7	ST		0.9
**9**	Right	Anterior Segment Dysgenesis Sclerocornea	ECCE	16.5	Aphakic	BV350	ST		0.42
**10**	Left	Corneal Scar	Corneal laceration repair Strabismus surgery ECCE Iris prosthetic implant	8.4	PCIOL	BV350	ST		6.9

Ten eyes of 7 patients had undergone previous glaucoma surgeries (Table 1, 2.0 ± 1.5 surgeries/eye, range 1-4): contact transcleral cycloablation (CTLC; 6 eyes of 4 patients), trabeculotomy (3 eyes of 2 patients), and Ahmed FP7 glaucoma valve implantation (1 eye of 1 patient). Four eyes of four patients had previously undergone cataract extraction, with three eyes being left aphakic and one receiving an intraocular lens implant. One eye had undergone a ruptured globe repair, strabismus surgery, and an artificial iris implantation for cosmesis.

Preoperatively, 7 patients (10 eyes) were less than 3 years of age and unable to cooperate for quantitative visual testing. Eight of these eyes were reactive to light and 2 could fix and follow. The remaining 4 eyes of 3 patients that were old enough for optotype acuity ranged from light perception to 20/125 (Table 2). All eyes had central corneal opacification, which prevented clear visualization of the anterior chamber or posterior segment. Mean preoperative IOP was 31.9 ± 8.9 mmHg (range 21-48 mmHg) on an average of 2.5 ± 1.2 (range 0-5) topical and/or oral glaucoma medications. Preoperatively, 3 eyes of 2 patients had clear crystalline lenses, 1 eye of 1 patient was pseudophakic, 3 eye of 2 patients had a cataractous lens, 3 eyes of 3 patients were surgically aphakic, and 4 eyes of 2 patients were congenitally aphakic.

**Table 2:** Preoperative and Final Examination Findings. Ocular findings at preoperative and final examinations of the 14 eyes of 10 patients which underwent combined endoscopic vitrectomy with posteriorly placed glaucoma drainage device

**Pt**	**Eye**	**Preop BCVA**	**Preop IOP (mmHg)**	**Preop Glaucoma Medications**	**Final BCVA**	**Final IOP (mmHg)**	**Final Glaucoma Medications**
**1**	Right	20/600	26	5	HM	8	0
	Left	LP	44	3	HM	10	0
**2**	Right	RTL	48	2	20/125	11	2
	Left	RTL	39	2	20/125	12	2
**3**	Left	RTL	33	2	RTL	15	1
**4**	Right	RTL	28	3	20/800	15	2
	Left	F&F	41	3	20/1600	15	0
**5**	Right	RTL	24	0	LP	21	2
**6**	Right	F&F	24	1	RTL	14	0
**7**	Right	RTL	23	3	F&F	10	0
	Left	RTL	23	3	F&F	10	0
**8**	Right	RTL	37	2	RTL	13	0
**9**	Right	20/200	21	3	20/300	12	3
**10**	Left	20/125	36	3	CF	18	0

*Pt* Patient, *BCVA* Best Corrected Visual Acuity, *LP* Light Perception, *RTL* Reactive to Light, *F&F* Fix and Follow, *HM* Hand Motions, *CF* Count Fingers, *IOP* Intraocular Pressure

Mean age at time of surgery was 4.61 ± 5.75 years (median 2.4, range 0.08-16.41) and the mean time of follow-up was 3.7 ± 2.4 years (median 4.0, range 0.4-6.9). Implanted GDDs included Ahmed FP7 (2 eyes of 2 patients), Ahmed FP8 (2 eyes of 1 patient), BV250 (4 eyes of 3 patients), and BV350 (6 eyes of 5 patients). All plates were positioned in the superotemporal quadrant, with the exception of one Ahmed FP7 which was placed superonasally due to scleral thinning at the site of a previous trabeculotomy. Six eyes of 3 patients underwent concurrent lens extraction and were left aphakic. One eye had cryotherapy for a vitreous tag secondary to persistent fetal vasculature. One eye had explantation of an encapsulated AGV.

**Fig. 1:**
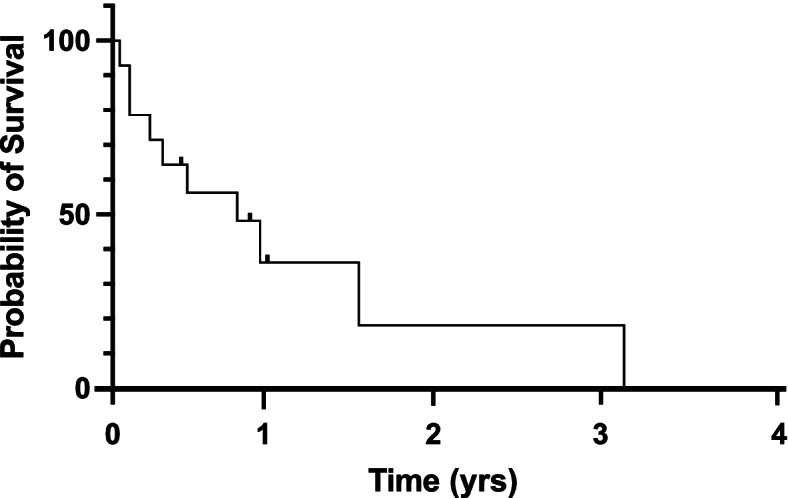
Survival curve for success of posteriorly-placed glaucoma drainage device placement (IOP control without glaucoma medications). Kaplan-Meier analysis demonstrated 1- and 2-year survival rates of 36% with 95% CI[10, 63] and 18% with 95% CI[1, 51], respectively

Four eyes (29%) of 3 patients were successful at time of final follow-up, while 10 eyes (71%) of 7 patients required at least 1 additional surgery (2.4 ± 1.3 additional surgeries/eye, range 1-5, median 2) for glaucoma or hypotony. Kaplan-Meier analysis demonstrated 1- and 2-year survival rates of 36% with 95% CI[10, 63] and 18% with 95% CI[1, 51], respectively (Fig 1). Importantly, the 4 eyes which remained successful, had a mean follow-up (0.7 ± 0.2 years, range 0.4-0.9 years, median 0.8 years) that was significantly shorter (*p*=0.0003) than the other 10 eyes (mean 4.9 ± 1.6 years, range 2.2-6.9 years, median 4.5 years). Seven eyes of six patients required additional glaucoma surgery for increased IOP (Table 3). The average length of time to next surgery was 9.6 ± 11.9 months (range 0.5-35.7, median 5.2). Three eyes of three patients underwent subsequent CTLC. One eye required CTLC followed by endoscopic cyclophotocoagulation (ECP). One eye had a blockage of the Baerveldt tube by vitreous and required revision. Three eyes of 3 patients failed due to hypotony requiring additional surgery (Table 3). One eye (patient 1, right eye) had a history of 360º CTLC over multiple treatments prior to endoscopic vitrectomy/BV350 placement. Subsequent surgeries were required due to alternating hypotony and increased IOP. The BV350 was first exchanged for a BV250, which was then replaced with an Ahmed FP8. However, the eye was still hypotonous such that the Ahmed FP8 was permanently ligated and eventually removed due to epithelial downgrowth, and the eye ultimately became phthisical. One eye (patient 4, left eye) had staged BV350 implantation with insertion of the tube in the pars plana 4 weeks after the plate was placed. The eye was hypotonous such that at 2 weeks after the second stage a temporary polyglactin ligature was placed. The intraocular pressure has been well-controlled since that ligature dissolved. The third eye (patient 6, right eye) developed hypotony after multiple penetrating keratoplasties and chronic retinal detachment which required removal of the tube 1.4 years after implantation.

**Table 3:** Surgical Outcomes. Surgical success, post-operative complications and additional intraocular surgeries in the 14 eyes of 10 patients which underwent combined endoscopic vitrectomy with posteriorly placed glaucoma drainage device

**Pt**	**Eye**	**Failure**	**Cause of Failure**	**Complications**	**GDD Survival (months)**	**Additional Intraocular Surgeries**
**1**	Right	Y	Hypotony	Phthisis	1.2	Exchange BV350 for BV250 due to hypotony
						Removal of BV250 tube from eye due to hypotony
						Removal of BV250 plate with placement of FP8 for increased IOP
						Placement of permanent ligature on FP8 tube for hypotony
						Removal of FP8 plate due to epithelial downgrowth
	Left	Y	Increased IOP	Tube Blockage	1.2	Flush of BV250 tube due to blockage
**2**	Right	Y	Increased IOP		8.7	Placement of BV250 inferonasally due to increased IOP
						CTLC x2 for increased IOP
						ECP for increased IOP
	Left	Y	Increased IOP	Retinal Detachment	2.6	Placement of FP7 superonasally due to increased IOP
						Flush of BV250 tube due to blockage
**3**	Left	Y	Increased IOP	Chronic Corneal Graft Failure	10.3	CTLC x 2 due to increased IOP
						ECP due to increased IOP
						PKP x 3 for corneal graft failure
**4**	Right	Y	Increased IOP		3.5	CTLC x 2 due to increased IOP
	Left	Y	Hypotony		0.5	Placement of temporary ligature on BV350 tube due to hypotony
**5**	Right	Y	Increased IOP		5.2	CTLC x 2 due to increased IOP
						Exchange trimmed FP7 for full size plate FP7 due to increased IOP
**6**	Right	Y	Hypotony	Retinal Detachment Chronic Corneal Graft Failure	17.2	Removal of BV250 tube from eye due to hypotony from retinal detachment
						Removal of BV250 plate due to extrusion
						PKP x 3 for corneal graft failure
**7**	Right	N			9.5	None
	Left	N			9	None
**8**	Right	N			10.8	None
**9**	Right	N			5	None
**10**	Left	Y	Increased IOP	Band keratopathy	35.7	CTLC x 1 due to increased IOP

At final follow-up, 1 eye was phthisical, however, IOP control was obtained in the remaining 13 eyes such that final IOP (13.1 ± 3.5 mmHg, range 8-21 mmHg) and number of glaucoma medications (0.9 ± 1.1 , range 0-3) were significantly decreased (*p*<0.0001) compared to preoperative values. A total of 4.4 ± 2.4 surgeries/eye (range 1-10, median 4) were performed to obtain pressure control. The most successful approach (8 of 14 eyes, 57%) combined cycloablation either prior to (5 eyes) or following (3 eyes) endoscopic vitrectomy and posteriorly-placed GDD. Eleven eyes showed improved (7 eyes of 4 patients) or stable (4 eyes of 4 patients) visual acuity at final follow-up. Three eyes had worse vision due to phthisis, chronic graft failure, and band keratopathy.

## Discussion

In children, corneal opacification, whether it be caused by congenital anomalies or trauma, is commonly associated with glaucoma [19]. These patients present unique challenges as obtaining IOP control often requires surgical management. In these eyes, the anatomy often makes it difficult to place a GDD within the anterior segment, such that the best option may be a posteriorly placed GDD combined with vitrectomy [5, 6]. However, in these eyes limited view from the corneal opacification can preclude standard wide-angle vitrectomy, requiring an endoscopic approach. While endoscopic vitrectomy and GDD placement has been described in adult eyes, there is minimal information in the literature regarding the pediatric population [12, 13]. We present, to the best of our knowledge, the largest study of children undergoing this combined approach.

In the two previously published studies in adult eyes, not surprisingly the underlying diagnoses differed from our pediatric cohort [12, 13]. In Tarantola et al, the majority of the 19 eyes (68%) had corneal opacification due to corneal graft failure while the remainder had corneal edema or scar, band keratopathy, or fibrosed pupil. Unfortunately, there was minimal information as to the original etiology or indication for the corneal transplants [12]. In Shaikh et al, 9 of the 13 eyes (69%) had corneal opacification secondary to aniridia, while the remainder had chemical burns, trauma, and Axenfeld-Rieger syndrome [13]. While many patients with aniridia gradually develop corneal opacification from pannus, this is not a common cause in children [20].

In our study, there was a range of etiologies; however, all of the eyes had complex anterior segment pathology with 93% having a history of prior intraocular surgery and 71% having previous glaucoma surgery. Sclerocornea with or without microphthalmia (43%) and Peters anomaly (29%) were the most common diagnoses in our cohort. While there is minimal information regarding the incidence and treatment of glaucoma in sclerocornea, 50-70% of eyes with Peters Anomaly develop glaucoma, of which more than half require IOP-lowering surgery [21, 22]. In both of these diseases, glaucoma is due to a combination of angle closure and abnormal aqueous humor outflow pathways such that anti-ocular hypertensive medications and angle surgery are less likely to control IOP. Consistent with this, we previously showed that GDDs yielded the highest success rate in Peters Anomaly [22]. Three of the eyes in our cohort had corneal decompensation due to prolonged elevated IOP from primary congenital glaucoma or glaucoma following cataract surgery. Although angle surgery is often successful in childhood glaucomas, eyes with severe anterior segment dysgenesis often require multiple surgeries to obtain IOP control [22, 23]. At least in the United States, in regards to trabecular meshwork bypass surgeries, GDDs have been favored over trabeculectomy with anti-fibrotics in infants and young children due to lower long-term risk of bleb-related infections [24, 25]. However, GDD implants may not be readily available in many parts of the world and also carry with them hardware risks such as exposure and tube retraction. Transcleral cycloabalation is also often utilized in eyes with corneal opacification, especially if there is limited access to endoscopic vitrectomy [26]. Nonetheless, it is important to realize that in complex eyes such as these that have minimal to no aqueous outflow, there is a fine line between glaucoma and hypotony. Using cycloablation, especially as the primary glaucoma procedures may ultimately result in phthisis [27].

Success of the combined endoscopic vitrectomy and posteriorly-placed GDD approach in adults, such that additional glaucoma surgeries were not required, was 74% in Tarantola et al and 100% in Shaikh et al. [12, 13] It is necessary to consider the differences in mean follow-up times in these previous published studies (62 months in Tarantola et al vs. 16 months in Shaikh et al). [12, 13] Despite obtaining IOP control in 93% of the eyes at final follow-up, the success rate in our pediatric cohort was under 36%. However, there was a significant difference in follow-up time as the 10 eyes that failed had significantly longer average follow-up (59 months) compared to the eyes which had remained successful (9 months). Certainly, longer follow-up of the 13 eyes in Shaikh et al as well as the successes in our cohort may show an increase in failure rate over time. Nevertheless, our success rate in children is lower than adults and this may be due to the complexity of the diseases and variability of prior glaucoma surgeries.

The failures can be divided into 2 groups: increased IOP requiring additional surgery (7 eyes) and hypotony (3 eyes). Of the 7 eyes with increased IOP, 1 had blockage which only required flushing of the tube while all of the others underwent placement of an additional GDD (3 eyes) and/or cycloablation (5 eyes). Five of these 7 eyes had prior glaucoma surgery, although cycloablation had been performed previously in the 1 eye that required a tube flush. An additional 5 eyes (6 total) had prior cycloablation, of which 3 eyes had continued IOP control after the endoscopic vitrectomy-GDD surgery. However, caution should be taken as extensive cycloablation prior to GDD placement may lead to either prolonged or temporary hypotony. This was the case in 2 of the eyes which failed due to hypotony, with one eventually becoming phthisical and the second requiring temporary re-ligation of the tube. Nevertheless, it is important to note that IOP control was obtained in 8 of the 14 eyes (57%) at final follow-up with a combination of the GDD placed concurrent with the endoscopic vitrectomy and either prior or sequential cycloablation. This parallels our case series of eyes with Peters Anomaly, suggesting that the combination of GDD and cycloablation is often required in complex pediatric eyes [22].

Additional complications, which were encountered included chronic corneal graft failure (2 eyes) and retinal detachment (2 eyes). It is well established that corneal transplantation has a lower success rate in children than adults. This is attributed to a more robust inflammatory response in young children and limbal stem cell deficiency, which is often present in sclerocornea and Peters Anomaly [28–30]. Both of the eyes in our study with chronic graft failure underwent multiple penetrating keratoplasties and eventually additional transplantation surgery was not pursued due to poor visual prognosis. This also underlies our decision for an endoscopic vitrectomy rather than utilizing a temporary keratoprosthesis followed by penetrating keratoplasty. Additionally, retinal detachment, which occurred in 2 eyes, is a post-operative concern especially following vitrectomy. This has been an argument against posteriorly placed tubes, although our previous study showed no increased risk compared to anteriorly placed tubes [5, 6]. Eyes with severe congenital anomalies often have abnormal transition from retina to pars plana and in some cases a complete lack of the pars plana [22]. This necessitates anterior placement of the trocars at or just posterior to the limbus, which can make the vitrectomy more challenging. However, the use of the endoscope allows for better visualization of the peripheral retina and ora serrata to assess if any treatment is needed for iatrogenic retinal breaks [9–11].

Taken together, we present the largest cohort to date of pediatric patients who underwent endoscopic vitrectomy with GDD placement. Limitations of our study include a small sample size, retrospective nature, lack of control population, varying disease etiology and severity, and relatively short follow-up time. A prospective study with potentially greater uniformity of eye pathologies is difficult due to the rarity of these diseases. Although we encountered complications and a high rate of reoperation, these eyes represent a significant challenge for ophthalmologists.

## Conclusions

The visual prognosis in pediatric eyes with corneal opacification is especially poor when coupled with glaucoma. The inability to obtain IOP control not only causes further vision loss, but can lead to pain and disfigurement. Although these eyes often require multiple glaucoma surgeries, combined endoscopic vitrectomy with posteriorly-placed GDD is a viable surgical option to establish aqueous outflow, achieve IOP control, and preserve vision.

## Supplementary Information

**Additional file 1:** **Supplemental Table 1.** Glaucoma Drainage Device Information.

## Data Availability

The datasets used and/or analyzed during the current study are available from the corresponding author (BLB).

## Abbreviations

GDD: glaucoma drainage device
IOP: intraocular pressure
BCVA: best corrected visual acuity
CTLC: contact transscleral laser cyclophotocoagulation
ECP: endoscopic cyclophotocoagulation
