# Supplementary material for: Outcomes of combined endoscopic vitrectomy and posteriorly placed glaucoma drainage devices in pediatric patients
Source: BMC Ophthalmol. 2022 Apr 1;22:149. doi: 10.1186/s12886-022-02373-3 (PMC8976343; doi:10.1186/s12886-022-02373-3)
Supplement: Supplementary file 1 — Additional file 1: Supplemental Table 1. Glaucoma Drainage Device Information. [file 12886_2022_2373_MOESM1_ESM.pdf]

Supplemental Table 1: Glaucoma Drainage Device Information

	BV350	BV250	Ahmed FP7	Ahmed FP8
**Anterior-Posterior Plate Length (mm)**	16	14	16	11
**Estimated Minimal Axial Length (mm) if GDD Placed in Superotemporal Quadrant**	20	18	20	16
**Plate Surface Area (mm^2)**	350	250	184	102
**Valved**<br>- **Immediate pressure lowering effect**<br>- **Less risk of post-operative hypotony**<br>- **Higher risk of hypertensive phase/ plateencapsulation**	No	No	Yes	Yes
**Non Valved**<br>- **Delayed pressure lowering effect (3-6 wks)**<br>- **Requires temporary ligation or staged procedure**<br>- **Greater risk of post-operative hypotony**<br>- **Lower risk of hypertensive phase/ encapsulation**	Yes	Yes	No	No

Comparison of characteristics of Baerveldt (BV) and Ahmed glaucoma drainage devices (GDD). Estimated minimal axial length based on anterior-posterior plate length, corneal diameter, and GDD plate placement at least 6 mm posterior to the limbus and more than 3 mm from the optic nerve. (Chandramouli SA et al, Reference #18).
